# Semantic Clustering during Verbal Episodic Memory Encoding and Retrieval in Older Adults: One Cognitive Mechanism of Superaging

**DOI:** 10.3390/brainsci14020171

**Published:** 2024-02-08

**Authors:** Clare Shaffer, Joseph M. Andreano, Alexandra Touroutoglou, Lisa Feldman Barrett, Bradford C. Dickerson, Bonnie Wong

**Affiliations:** 1 Department of Psychology, College of Science, Northeastern University, Boston, MA 02115, USA; l.barrett@northeastern.edu; 2 Department of Psychiatry, Massachusetts General Hospital and Harvard Medical School, Boston, MA 02114, USA; jandreano@mgh.harvard.edu (J.M.A.); brad.dickerson@mgh.harvard.edu (B.C.D.); 3 Athinoula A. Martinos Center for Biomedical Imaging, Massachusetts General Hospital and Harvard Medical School, Boston, MA 02129, USA; atouroutoglou@mgh.harvard.edu; 4 Frontotemporal Disorders Unit, Massachusetts General Hospital and Harvard Medical School, Boston, MA 02129, USA; 5 Alzheimer’s Disease Research Center, Massachusetts General Hospital and Harvard Medical School, Boston, MA 02129, USA; 6 Department of Neurology, Massachusetts General Hospital and Harvard Medical School, Boston, MA 02114, USA

**Keywords:** aging, strategy use, memory, superaging

## Abstract

Normal aging is commonly accompanied by a decline in cognitive abilities, including memory, yet some individuals maintain these abilities as they get older. We hypothesize that semantic clustering, as an effective strategy for improving performance on episodic recall tasks, may contribute to the maintenance of youthful memory in older adults. We investigated the dynamics of spontaneous production and utilization of the semantic clustering strategy in two independent samples of older adults who completed a list learning paradigm (N_1_ = 40 and N_2_ = 29, respectively). Specifically, we predicted and observed that older adults who spontaneously used a semantic clustering strategy throughout the encoding process learned more words by the culmination of the encoding trials (Sample 1, *R*^2^ *=* 0.53, *p* < 0.001; Sample 2, *R*^2^ *=* 0.51, *p* < 0.001), and that those who utilized this strategy during retrieval recalled more words, when compared to older adults who did not produce or utilize a semantic clustering strategy during both a short (Sample 1, *R*^2^ = 0.81, *p* < 0.001; Sample 2, *R*^2^ = 0.70, *p* < 0.001) and long delay retrieval (Sample 1, *R*^2^ = 0.83, *p* < 0.001; Sample 2, *R*^2^ = 0.77, *p* < 0.001). We further predicted and observed that older adults who maintained a youthful level of delayed free recall (i.e., “Superagers”) produced (Sample 1, F(1, 38) = 17.81, *p* < 0.0001; Sample 2, F(1, 27) = 14.45, *p* < 0.0001) and utilized (Sample 1, F(1, 39) = 25.84, *p* < 0.0001; Sample 2, F(1, 27) = 12.97, *p* < 0.01) more semantic clustering than did older individuals with normal memory for their age. These results suggest one cognitive mechanism through which Superagers maintain youthful memory function and raise the possibility that older adults may be able to train themselves to use strategies to promote better memory.

## 1. Introduction

Aging is often accompanied by a decline in memory, even in healthy individuals [1], yet there is considerable heterogeneity in the steepness of this decline [2,3]. Some individuals show a very steep drop in memory as they age, while others show a more gradual decline. Still others see very little decline, maintaining a level of memory performance commensurate with middle-aged [4,5,6,7] and even young adulthood [8,9,10]. Older adults with “youthful” memory abilities have been referred to as “Superagers” [4,5,6,7,8,9,10,11], a category of older adult which has been identified in previous studies across multiple populations, and defined by several measures, including performance on episodic delayed free recall memory tasks. Tasks of this sort permit differential use of strategies that contribute to memory encoding and retrieval abilities [12,13,14,15], which may help explain the wide variation in performance seen in older adults. That is, typical older adults may not remember as much information they study relative to younger adults or Superagers, in part because they may not spontaneously organize material (unsupported intentional encoding) [16,17,18]. Behavioral studies suggest that certain cognitive strategies improve memory performance, whether the strategy is used spontaneously [12,19,20,21] or after explicit instruction [22,23]. Strategies vary, ranging from simple rehearsal (i.e., repetition of stimuli in the phonological loop) to those that are more cognitively demanding, such as semantic clustering, which involves reorganizing stimuli according to meaning or a shared semantic category.

Research that studies cognitive strategies and their impact on memory distinguish between understanding the existence of strategy and actually using it, referred to as “production” and “utilization”, respectively [19,24,25]. Strategy production refers to the ability to spontaneously recognize that there is a strategy available for organizing information to be remembered, while utilization of that strategy refers to the ability to actually use the strategy to improve memory performance. Correspondingly, a production deficiency refers to a diminished ability to spontaneously identify the opportunity to apply a strategy (without an explicit cue/instruction) and a utilization deficiency refers to the failure to use a known strategy to improve memory, i.e., where participants receive training on strategies which aid memory and show evidence of being able to use it, but fail to do so even when explicitly instructed (e.g., [26]).

One possible source of heterogeneity in the memory performance and aging literature may stem from differential strategy use during either the learning and/or retrieval phases of a memory task. For example, in neurotypical adults, semantic clustering has been reported as an effective strategy for improving performance on episodic recall for list-learning paradigms like the California Verbal Learning Test (CVLT) and the Hopkins Verbal Learning Test (HVLT) [27,28,29]. Studies of strategy use in older adults suggest that the use of active strategies like semantic clustering is mixed. Some studies suggest that strategy production and use declines with age [14], whereas other studies report no age-related differences (e.g., [30,31]). Those who do produce and use active strategies exhibit improvements in memory performance, however [12,32].

In many list-learning memory paradigms such as the CVLT, the task is split into several “phases”: first an “encoding phase” in which subjects are read a list of words, and immediately prompted to repeat as many words back to the experimenter as possible. This process repeats for five trials, and is referred to as “Immediate Free Recall” trials [33]. Performance on the first encoding/recall trial relies heavily on working memory [34], although some individuals demonstrate nearly immediate spontaneous use of a strategy to reorganize material based on semantic relationships [35,36]. Over the course of repeated encoding trials, semantic associations and executive control may be increasingly employed to organize the material [13,21,34]. After a “distractor” list is introduced, subjects are asked to complete the “short delayed free recall” (SDFR) phase of the task, in which they are prompted to spontaneously recall as many words from the original list as possible. After an additional twenty-minute delay, they are then asked to recall the list after being explicitly given semantic category cues, before completing the “long delayed free recall” (LDFR) phase, where they are prompted to spontaneously recall as many words from the original list as possible. While a substantial number of studies have examined strategy production during encoding as it relates to delayed retrieval performance (e.g., [12,19,20,21]), there is little data focusing on the production of a strategy during the encoding phase to directly improve performance on the encoding trials themselves and how this impacts recall on subsequent retrieval trials. Consequently, it is unclear if older adults can identify the opportunity to apply a strategy to improve their performance during the process of learning the material, and what bearing this has on performance during the retrieval portion of the task.

In the present study, we investigated older adults’ use of semantic clustering during the performance of the CVLT, expanding on previous work on strategy production and utilization [24,25,26] by developing a method for evaluating the spontaneous production of semantic clustering during encoding and the spontaneous continued utilization of that strategy during short and long-delayed retrieval. We hypothesized that older adults who actively produce a semantic clustering strategy throughout the encoding process will have learned more words by the culmination of the encoding trials, and that those who utilize that strategy during both uncued (short-delay) and cued (long-delay) retrieval will recall more words than those who do not produce or utilize a semantic clustering strategy. We further hypothesized that this is one of the cognitive mechanisms that enables some older adults to perform at a youthful level on delayed free recall, which is the primary measure typically employed to define “Superaging”.

## 2. Materials and Methods

### 2.1. Participants

Two independent samples of community-dwelling healthy older adults were recruited from the greater Boston area to participate in a longitudinal study as part of our Massachusetts General Hospital Brain Resilience in Aging: Integrated Neuroscience Studies (BRAINS) program. Sample 1 included 40 adults (*Mean_Age_* = 66.87, *SD_Age_* = 5.48, range = 60–81 years, 18 women, 22 men). Sample 2 included 29 adults (*Mean_Age_* = 69.57, *SD_Age_* = 6.94, range = 60–86 years, 18 women, 11 men). All participants were right-handed native English speakers with normal or corrected-to-normal vision and hearing and with no history of substance abuse, neurological disorder, or psychiatric disorder and who were not taking psychoactive medications. Additionally, all participants scored within 1.5 standard deviations of published normative values on all neuropsychological screening tests. The experimental protocols involving human subjects were approved by the Mass General Brigham Healthcare System Institutional Review Board (Sample 1: 2012P001671, active on 12 September 2012; Sample 2: 2007P000622, active on 2 May 2007). All experiments were undertaken with the understanding and written consent of each participant.

For some analyses, we included all individuals from each sample. For others, we separated Superagers from typical older adults. As we have previously published (e.g., [10]), we defined Superagers as those older participants who performed at or above the mean for young adults (ages 18–32) on the long delay free recall measure of the CVLT [33] and no lower than 1 standard deviation below the mean for their age group on the Trail Making Test Part B (TMT-B) [37].

### 2.2. California Verbal Learning Test (CVLT) and Chance-Adjusted Semantic Clustering Score

Participants visited the laboratory and completed the CVLT [33], a 16-item verbal list-learning test that includes 5 encoding trials followed by short-delay and long-delay free and cued recall and recognition. A trained staff member guided the participants through the test and was responsible for keeping a record of the participant’s responses for each trial.

A semantic clustering score was calculated using the formula described in the test manual [33], was a measure of strategy production during the encoding trials of the CVLT, and was used as a measure of strategy utilization during the retrieval trials of the CVLT. The semantic clustering score is calculated by first computing an “observed semantic clustering score” (one point every time a word is recalled adjacent to another word belonging in the same category). Then, a “chance-expected” score is calculated based on the number of words recalled, which is then used to compute a “chance-adjusted” score to account for higher semantic clustering scores only because a greater number of words were recalled [38]. Higher semantic clustering scores reflect an increased number of words from the same category being sequentially recalled and scoring accounts for the number of categories that are being clustered. Chance-adjusted semantic clustering scores range from 9, representing full semantic organization when all 16 words are fully clustered in all 4 categories, to −3, which represents no semantic clustering when all 16 words are recalled. A positive score represents the degree to which words are recalled in order based on semantic categories above chance level, and a negative score represents the degree of semantic categorization below chance level [38]. The chance-adjusted semantic categorization scores, as well as recall scores (i.e., total number of words recalled per trial) from the encoding trials and the short-delay free recall trial were used in the primary analyses as predictors and outcome variables. The long-delay free recall trial of the CVLT, which takes place later in the test after a 20 min delay, was examined separately because it is typically used in defining Superagers, but since it occurs after a category-cued recall trial, we report it here as an additional analysis, recognizing that participants have been given an explicit cue to the semantic structure of the list. Our aim is to study both uncued and cued strategy utilization, while production of the strategy itself during the learning process is uncued.

### 2.3. Hypotheses and Data Analysis

#### 2.3.1. Hypothesis 1. The Production of a Semantic Clustering Strategy Will Improve Encoding

The outcome variable of interest for Hypothesis 1 was the total number of words learned by the fifth and final trial of the encoding trials (Trial 5 recall). In order to account for the influence of rote working memory on encoding [34] in addition to the production of a semantic clustering strategy, we included Trial 1 recall and the chance-adjusted semantic clustering score on Trial 5 as predictors in our model. A general linear regression model was computed to evaluate the relationships between the independent variables (Trial 1 recall and Trial 5 semantic clustering score) and the dependent variable (Trial 5 recall). All data analysis was performed in RStudio (2020). For all analyses, age, sex, and years of education were entered into a Pearson correlation (for age and years of education) and point-biserial correlation (for sex) to assess relationships with both predictors and outcome variables from each model. Additionally, all demographic variables were entered as additional predictors in each model to assess the influence of these variables on the outcome. Variance inflation factor (VIF) and tolerance values were computed for all predictor variables to assess for multicollinearity (VIF values greater than 5 and tolerance values less than 0.1 indicated problematic multicollinearity; [39]). Finally, we performed Shapiro–Wilk and Levene’s tests to ensure that our data did not violate the assumptions of our statistical models and found no violations in either sample. For all analyses, alpha was set at 0.05, and we analyzed each sample independently to determine whether effects of primary interest replicated across the two samples. We then compared the model effects from the two samples to assess for heterogeneity and then used meta-analytic procedures for combining effect sizes and significance tests described in Chapter 4 of Rosenthal [40] to summarize the results across both studies.

#### 2.3.2. Hypothesis 2. The Utilization of a Semantic Clustering Strategy Will Improve Short- and Long-Delay Free Recall

To assess how the utilization of a strategy influenced recall on the short delay trial, we used the total number of words recalled during the short-delay free recall (SDFR) trial of the CVLT as our outcome variable. In this analysis, we sought to investigate how (1) Trial 5 recall (as examined in Hypothesis 1), and (2) the utilization of the semantic clustering strategy during the SDFR retrieval trial affected the outcome variable. A general linear regression model was computed to evaluate the relationships between the independent variables (Trial 5 recall and chance-adjusted semantic clustering score during the SDFR trial) and the dependent variable (recall on the SDFR trial). To assess how utilization of a strategy influenced recall on the long-delay trial, we used the total number of words recalled during the long-delay free recall (LDFR) trial of the CVLT as our outcome variable. In this analysis, we aimed to investigate how (1) SDFR recall and (2) the utilization of the semantic clustering strategy during the LDFR trial affected the outcome variable. As in the first analysis, a general linear regression model was computed to evaluate the relationships between the independent variables (SDFR and chance-adjusted semantic clustering score during the LDFR trial) and the dependent variable (LDFR recall). For all analyses, age, sex, and years of education were entered into a Pearson correlation (for age and years of education) and point-biserial correlation (for sex) to assess relationships with both predictors and outcome variables from each model. Additionally, all demographic variables were entered as additional predictors in each model to assess the influence of these variables on the outcome. VIF and tolerance values were again computed for all predictor variables to assess for multicollinearity. Finally, we analyzed each sample independently to determine whether effects of primary interest replicated across the two samples, and then applied meta-analytic procedures for combining effect sizes and significance tests [40] to summarize the results across both studies.

#### 2.3.3. Hypothesis 3. Superagers Will Produce and Utilize a Semantic-Clustering Strategy during Encoding and Recall to a Greater Degree Than Typical Older Adults

We examined whether Superagers (SA) in each sample produced (i.e., higher semantic clustering scores during encoding) and utilized (i.e., higher semantic clustering scores during both short- and long-delay free recall) the semantic clustering strategy more so than their typical older adult (TOA) counterparts. The measures used in our models are orthogonal to the classification used for Superaging, as we are defining performance using semantic clustering on both short and long-delay retrieval trials. We computed two separate models using one-way analysis of variance to evaluate the relationships between Group (SA versus TOA) and both production and utilization of the semantic clustering strategy. Additionally, we wanted to assess whether the utilization of semantic clustering during the long-delay free recall trial was more prevalent in SA versus their TOA counterparts, so we compared the two groups using a one-way analysis of variance to look at semantic clustering during the long-delay free recall trial. We again analyzed each sample independently to determine whether effects of primary interest replicated across the two samples, and then applied meta-analytic procedures for combining effect sizes and significance tests [40] to summarize the results across both studies.

## 3. Results

### 3.1. Participant Characteristics

The first sample included 40 adults (*Mean_Age_* = 66.87, *SD_Age_* = 5.48, *range_Age_* = 60–81 years, 18 women, 22 men), and the second sample was made up of 29 adults (*Mean_Age_* = 69.57, *SD_Age_* = 6.94, *range_Age_* = 60–86 years, 18 women, 11 men) (see Table 1).

### 3.2. The Production of a Semantic Clustering Strategy Improves Encoding

**Figure 1:**
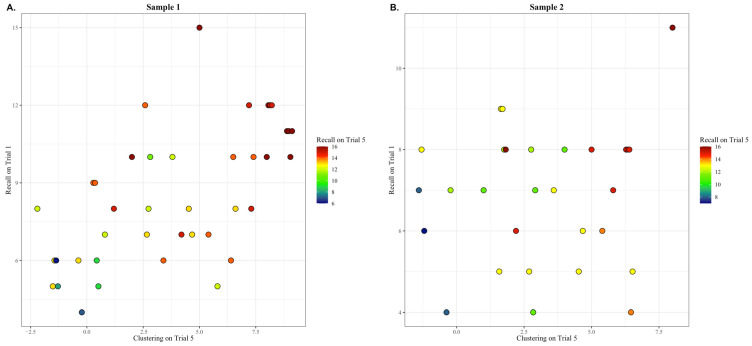
The spontaneous production of a semantic clustering strategy during encoding is associated with better learning (i.e., Trial 1 Recall and Trial 5 Semantic Clustering predict Trial 5 Recall). (**A**) In sample 1, individuals who scored higher on both Trial 1 encoding and Trial 5 semantic clustering strategy production had the highest Trial 5 Recall (variance in Trial 5 recall predicted by the independent variables was *R*^2^ = 0.53, *p* < 0.01). (**B**) In sample 2, the results supported a similar finding; individuals who scored higher on Trial 1 encoding and Trial 5 semantic clustering had the highest Trial 5 Recall (*R*^2^ = 0.51, *p* < 0.01). Individuals with the highest level of Trial 5 Recall are shown in red, those with a medium level of Trial 5 Recall are shown in yellow and green, and those with the lowest level of Trial 5 Recall are shown in blue.

Results from the general linear regression model using data from Sample 1 supported our hypothesis. The fitted regression model was Trial 5 learning = Intercept + X × (Trial 1 learning) + Y × (Trial 5 semantic clustering). The overall regression was statistically significant (*R*^2^ *=* 0.53, F(2, 37) = 23.34, *p* < 0.001). Trial 5 learning was predicted by both recall during Trial 1 (β = 0.42, *B* = 0.41, *p* < 0.01) and chance-adjusted semantic clustering during Trial 5 (β = 0.42, *B* = 0.30, *p* < 0.01; see Figure 1A). These results were replicated in Sample 2. Again, the overall regression was statistically significant (*R*^2^ = 0.51, F(2, 26) = 15.76, *p* < 0.001). Trial 5 recall was predicted by both recall during Trial 1 (β = 0.28, *B* = 0.39, *p* < 0.05) and chance-adjusted semantic clustering during Trial 5 (β = 0.65, *B* = 0.57, *p* < 0.001) (see Figure 1B). To compare the effect sizes of the model from each sample, we employed the meta-analytic procedure described above. The effects of the model did not differ significantly between the two samples, in either their size (*z* = 0.05, *p* = 0.48) or in their *p* values (*z* = 0.72, *p* = 0.75). The estimate of the combined effects across the two samples was 0.72. For Sample 1, age, sex, and education were not significant predictors of Recall on Trial 5; however, in Sample 2, we found that age was a significant predictor of recall on Trial 5 (β = −0.11, *p* < 0.05; see Table S3) and after inclusion of demographic variables in our model, recall on Trial 1 was no longer a significant predictor (see Table S3). However, clustering on Trial 5 remained a robust predictor of recall on Trial 5 across both samples, and tests to check for collinearity indicated that multicollinearity was not a concern (Sample 1: VIF = 1.59, Tolerance = 0.63, Sample 2: VIF = 1.01, Tolerance = 0.98).

**Figure 2:**
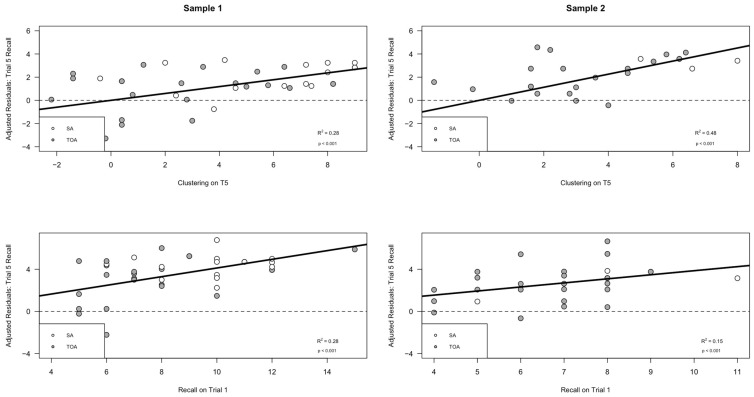
The spontaneous production of a semantic clustering strategy during encoding is associated with better encoding (i.e., Trial 1 Recall and Trial 5 Semantic Clustering predict Trial 5 Recall). This graph is a different depiction of the results in Figure 1, showing that better Trial 1 encoding and greater Trial 5 semantic clustering production each contributed independently to better Trial 5 encoding. (Sample 1, top graph) The *y* axis displays the adjusted residual variance in Trial 5 recall after the variance due to trial 1 learning has been removed; the scatterplot illustrates the relationship between adjusted Trial 5 recall and semantic clustering production during the 5th encoding trial. (Sample 1, bottom graph) The *y* axis displays the adjusted residual variance in Trial 5 recall after the variance due to the production of semantic clustering on Trial 5 was removed; the scatterplot illustrates the relationship between the adjusted Trial 5 recall and Trial 1 learning in Sample 1. (Sample 2, top graph) The *y* axis displays the residual variance in Trial 5 recall after the variance due to Trial 1 learning has been removed; the scatterplot illustrates the relationship between adjusted Trial 5 recall and semantic clustering production during the 5th encoding trial. (Sample 2, bottom graph) The *y* axis displays the residual variance in Trial 5 recall after the variance due to semantic clustering production was removed; the scatterplot illustrates the relationship between the adjusted Trial 5 recall and Trial 1 learning in Sample 2. SA = Superagers, hollow circles; TOA = typical older adult, filled circles.

The findings for our production model displayed a robust replication across both samples 1 and 2. To better understand the various contributions of each predictor (i.e., semantic clustering on Trial 5 and recall on Trial 1), we computed adjusted residual statistics and plots depicting the independent variance contributed by each predictor. The adjusted residual plots revealed some small differences across the two samples. The *R*^2^ values obtained from the adjusted production models indicated that Sample 1 participants tended to rely on both predictors in each model equally to improve performance, such that both semantic clustering (*R*^2^ = 0.282, *p* < 0.001) and Trial 1 recall (*R*^2^ = 0.281, *p* < 0.001) contributed equal amounts of explained variance in the combined model (see Figure 2, left panel). However, for Sample 2, a slightly different pattern emerged, where the proportion of variance explained by semantic clustering (*R*^2^ = 0.48, *p* < 0.001) was nearly double that explained by Trial 1 recall (*R*^2^ = 0.15, *p* < 0.001) (see Figure 2, right panel). These residual plots reinforce the idea that the overall model replicated fully across both samples, such that greater clustering and recall predicted increased Trial 5 recall; however, the contributions of each predictor showed small differences across Samples 1 and 2.

### 3.3. The Utilization of a Semantic Clustering Strategy Improves Short- and Long-Delay Free Recall

#### 3.3.1. Results from Semantic Clustering Strategy Utilization during Short-Delay Free Recall

**Figure 3:**
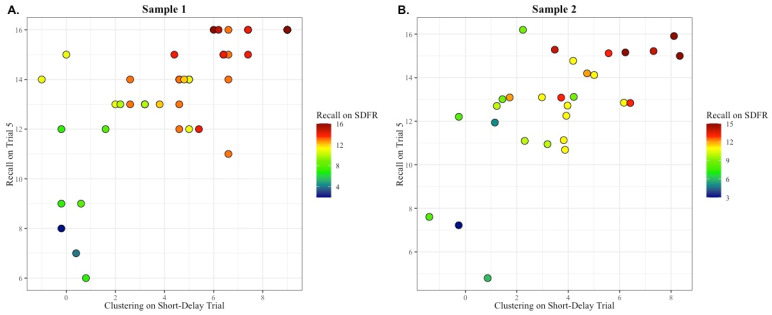
The spontaneous utilization of a semantic clustering strategy during retrieval is associated with better free recall (i.e., Short-Delay recall semantic clustering and Trial 5 recall predict Short-Delay recall performance). (**A**) In sample 1, individuals who scored higher on both Trial 5 recall and short-delay semantic clustering strategy utilization had the highest short-delay recall (variance in short-delay recall predicted by the independent variables was *R*^2^ = 0.81, *p* < 0.01). (**B**) In sample 2, the finding was again that high Trial 5 recall and short-delay clustering yielded high short delay free recall (*R*^2^ = 0.70, *p* < 0.01). Individuals with the highest level of short-delay free recall (SDFR) are shown in red, those with a medium level of SDFR are shown in yellow and green, and those with the lowest level of SDFR are shown in blue.

Results from the general linear model using data from Sample 1 supported our hypothesis that semantic clustering utilization improved recall performance during the short-delay retrieval trial. For clarity, this model and the results will be referred to as the “short-delay utilization model” to distinguish from the separate utilization model with long-delay free recall as the outcome variable, described below. For the short-delay utilization model, the fitted regression equation was Short-Delay Performance = Intercept + X × (Trial 5 recall + Y × (Short-Delay semantic clustering). The overall regression was statistically significant (*R*^2^ = 0.81, F(2, 37) = 83.14, *p* < 0.001). Retrieval Performance on the free recall trial was predicted by both Trial 5 recall (β = 0.52, B = 0.66, *p* < 0.001) and semantic clustering strategy utilization during the short-delay free recall trial (β = 0.46, B = 0.52, *p* < 0.001) (See Figure 3A). These results were replicated again in Sample 2. The overall regression was statistically significant (*R*^2^ = 0.70, F(2, 26) = 33.57, *p* < 0.001). As in Sample 1, retrieval performance on the free recall trial was predicted by both Trial 5 recall (β = 0.36, B = 0.40, *p* < 0.05) and semantic clustering utilization during the free recall trial (β = 0.57, B = 0.67, *p* < 0.001) (See Figure 3B).

As in the production analysis, we summarized across the two samples using the meta-analytic procedure described above to compare the effects of each model from the two samples. The effects of the model did not differ significantly between the two samples, in either their effect sizes (z = −0.86, *p* = 0.20) or in their *p* values (z = 1.61, *p* = 0.94). The estimate of the combined effects across the two samples was 0.79. Sex and education were not significant predictors of recall on the short-delay trial, although age was a significant predictor for Sample 1 only (β = −0.10, *p* < 0.05). Tests to check for collinearity indicated that multicollinearity was not a concern (Sample 1: VIF = 1.89, Tolerance = 0.52; Sample 2: VIF = 1.74, Tolerance = 0.57).

**Figure 4:**
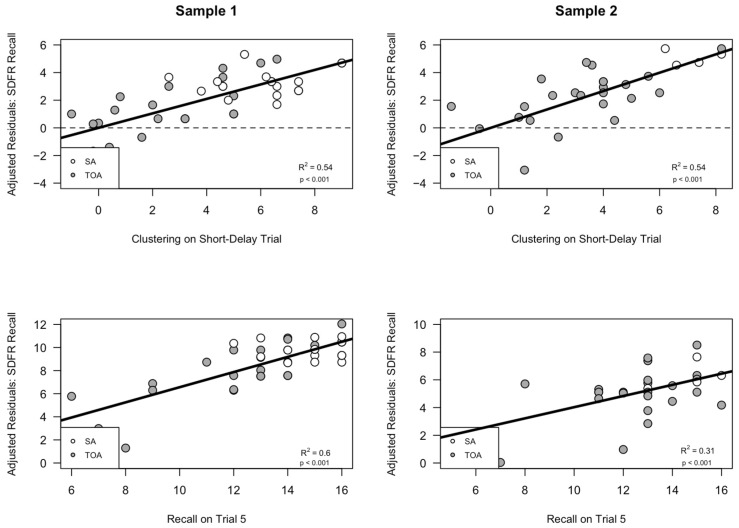
The spontaneous utilization of a semantic clustering strategy during retrieval is associated with better free short-delay free recall (i.e., Short-Delay recall semantic clustering and Trial 5 recall predict Short-Delay recall performance). This graph is a different depiction of the results in Figure 3, showing that better Trial 5 recall and greater short delay semantic clustering utilization each contributed independently to better short delay free recall. (Sample 1, top graph) The *y* axis displays the adjusted residual variance in short delay free recall after the variance due to recall on Trial 5 was removed; the scatterplot illustrates the relationship between the adjusted short delay recall and semantic clustering utilization in Sample 1. (Sample 1, bottom graph) The *y* axis displays the adjusted residual variance in short delay free recall after the variance due to semantic clustering score has been removed; the scatterplot illustrates the relationship between adjusted short delay recall and recall during the fifth encoding trial. (Sample 2, top graph) The *y* axis displays the residual variance in short delay free recall after the variance due to recall on Trial 5 was removed; the scatterplot illustrates the relationship between the adjusted short delay recall and semantic clustering utilization in Sample 2. (Sample 2, bottom graph) The *y* axis displays the residual variance in short delay free recall after the variance due to semantic clustering score has been removed; the scatterplot illustrates the relationship between adjusted short delay recall and recall during the 5th encoding trial. SA = Superagers, hollow circles; TOA = typical older adult, filled circles.

As in the production model, the findings from our utilization model for the short-delay free recall trial displayed a full replication across both samples 1 and 2. In following our protocol from the production models, we created similar adjusted residual plots that depicted the independent variance contributed by each predictor (i.e., Trial 5 recall and semantic clustering during the short delay recall trial). As in the production models, the adjusted residual plots showed small differences in the contributions of each predictor; interestingly, the pattern was the same as that seen in the production models. Specifically, the *R*^2^ values obtained from the adjusted short-delay utilization models indicated that Sample 1 participants tended to rely on both predictors in each model equally to improve performance, such that both semantic clustering (*R*^2^ = 0.54, *p* < 0.0001) and Trial 5 recall (*R*^2^ = 0.60, *p* < 0.0001) contributed near equal amounts of explained variance in the combined model (see Figure 4, left panel). However, for Sample 2, a slightly different pattern emerged, where the proportion of variance explained by semantic clustering (*R*^2^ = 0.54, *p* < 0.0001) was nearly double that explained by Trial 5 recall (*R*^2^ = 0.313, *p* < 0.0001) (see Figure 4, right panel). These residual plots reinforce the idea that the overall model replicated fully across both samples, such that greater clustering and recall predicted increased SDFR trial recall; however, the contributions of each predictor showed small differences across Samples 1 and 2.

#### 3.3.2. Results from Semantic Clustering Strategy Utilization during Long-Delay Free Recall

**Figure 5:**
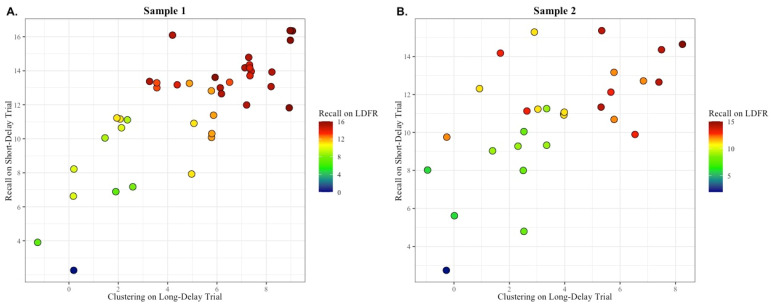
The spontaneous utilization of a semantic clustering strategy during retrieval is associated with better long-delay free recall (i.e., long-delay recall semantic clustering and short-delay recall predict long-delay recall performance). (**A**) In Sample 1, individuals who scored higher on both short-delay free recall and long-delay semantic clustering strategy utilization had the highest long-delay recall (variance in short-delay recall predicted by the independent variables was *R*^2^ = 0.83, *p* < 0.001). (**B**) In Sample 2, results suggested a similar finding, where individuals scoring higher on both short delay free recall and long delay semantic clustering utilization had high long-delay free recall (*R*^2^ = 0.77, *p* < 0.001). Individuals with the highest level of long-delay free recall (LDFR) are shown in red, those with a medium level of LDFR are shown in yellow and green, and those with the lowest level of LDFR are shown in blue.

As in the short-delay utilization model, results from the general linear model using data from Sample 1 supported our hypothesis that semantic clustering utilization improved recall performance during the long-delay retrieval trial. The fitted regression model was Long-Delay Recall Performance = Intercept + X × (Short-delay recall + Y × (Long-Delay semantic clustering). The overall regression was statistically significant (*R*^2^ = 0.83, F(2, 37) = 94.76, *p* < 0.001). Performance on the long-delay free recall trial was predicted by both short-delay recall (β = 0.70, B = 0.71, *p* < 0.001) and semantic clustering strategy utilization during the long-delay free recall trial (β = 0.25, B = 0.30, *p* < 0.001) (See Figure 5A). These results were replicated again in Sample 2. The overall regression was statistically significant (*R*^2^ = 0.77, F(2, 26) = 49.17, *p* < 0.001). As in Sample 1, performance on the long-delay free recall trial was predicted by both short-delay free recall (β = 0.64, B = 0.65, *p* < 0.05) and semantic clustering utilization during the long-delay free recall trial (β = 0.34, B = 0.40, *p* < 0.001) (See Figure 5B).

As in the production and short-delay utilization analysis, we summarized across the two samples using the meta-analytic procedure described above. The effects of the model did not differ significantly between the two samples, in either their effect sizes (z = 1.07, *p* = 0.14) or in their *p* values (z = 1.33, *p* = 0.90). The estimate of the combined effects across the two samples was 0.80. Age, sex, and education were not significant predictors of recall on the long delay trial and tests to check for collinearity indicated that multicollinearity was not a concern (Sample 1: VIF = 2.44, Tolerance = 0.41; Sample 2: VIF = 1.63, Tolerance = 0.61).

**Figure 6:**
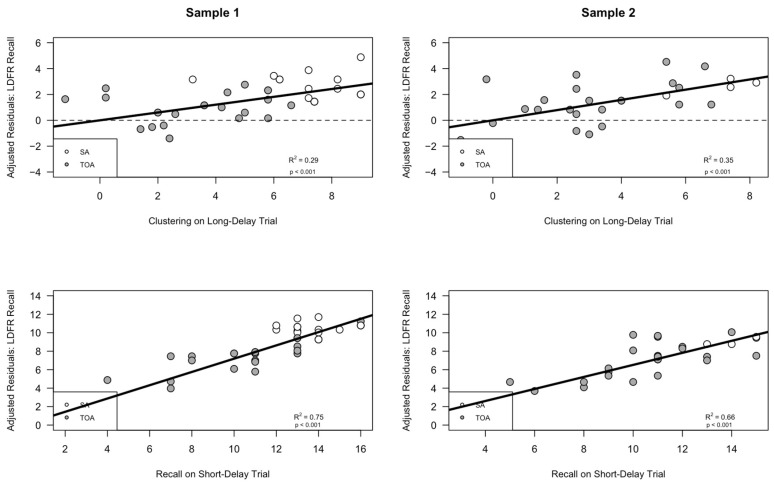
The spontaneous utilization of a semantic clustering strategy during retrieval is associated with better free long-delay free recall (i.e., long-delay recall semantic clustering and short-delay trial recall predict long-delay recall performance). This graph is a different depiction of the results in Figure 5, showing that better short-delay trial recall and greater long-delay semantic clustering utilization each contributed independently to better long delay free recall performance. (Sample 1, top graph) The *y* axis displays the adjusted residual variance in long delay free recall after the variance due to recall on the short-delay trial was removed; the scatterplot illustrates the relationship between the adjusted long delay recall and semantic clustering utilization in Sample 1. (Sample 1, bottom graph) The *y* axis displays the adjusted residual variance in long-delay free recall after the variance due to semantic clustering has been removed; the scatterplot illustrates the relationship between adjusted long delay recall and recall during the short-delay recall trial. (Sample 2, top graph) The *y* axis displays the residual variance in long delay free recall after the variance due to recall on the short-delay trial was removed; the scatterplot illustrates the relationship between the adjusted long-delay free recall performance and semantic clustering utilization in Sample 2. (Sample 2, bottom graph) The *y* axis displays the residual variance in short delay free recall after the variance due to semantic clustering score has been removed; the scatterplot illustrates the relationship between adjusted long-delay recall performance and recall during the short-delay trial. SA = Superagers, hollow circles; TOA = typical older adult, filled circles.

Similar to both the production and short-delay utilization model, the findings from our long-delay utilization model replicated across both samples 1 and 2. We again created adjusted residual plots that depicted the independent variance contributed by each predictor (i.e., short-delay free recall score and semantic clustering during the long-delay recall trial). As in the production and short-delay utilization models, the adjusted residual plots showed small differences in the contributions of each predictor; however, the pattern was consistent across both samples, and contrasted the patterns seen in prior models. Specifically, the adjusted *R*^2^ values obtained for each predictor from the long-delay utilization models indicated that for Sample 1 participants, recall on the prior short-delay trial was a stronger indicator of improved performance on the subsequent long-delay trial than semantic clustering utilization on that same long delay trial, such that short-delay recall (*R*^2^ = 0.75, *p* < 0.0001) was a greater predictor of long-delay recall than long-delay semantic clustering utilization (*R*^2^ = 0.30, *p* < 0.0001; see Figure 6, left panel). Interestingly, for Sample 2, the same pattern was replicated; where the proportion of variance explained by short-delay recall (*R*^2^ = 0.66, *p* < 0.0001) was nearly double that explained by long-delay semantic clustering score (*R*^2^ = 0.35, *p* < 0.0001; see Figure 6, right panel). These residual plots indicate that semantic clustering utilization during the long-delay trial may not be as crucial to memory performance as the preceding short-delay recall trial which, as we have shown in our short-delay utilization model, is bolstered by the utilization of semantic clustering strategy during that trial. Thus, the continued maintenance of the semantic-clustering strategy into the long-delay trial itself might not be as crucial to long-delay recall performance as the initial utilization of the strategy during short-delay free recall. One question that arises from this analysis is whether these patterns can be explained by the different levels of strategy utilization employed by Superagers versus typical older adults, a topic to which we now turn.

### 3.4. Superagers Produce and Utilize a Semantic-Clustering Strategy during Encoding and Recall to a Greater Degree Than Typical Older Adults

**Figure 7:**
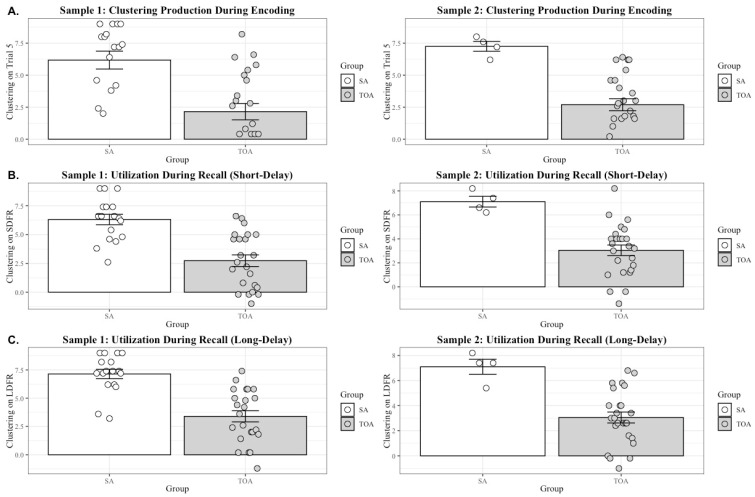
Superagers produce and utilize semantic clustering to a greater degree than typical older adults. (**A**) Production of semantic clustering strategy was defined by the semantic clustering score obtained during the fifth and final trial of encoding, depicted on the *y* axis. For both Sample 1 and Sample 2, the Superagers tended to produce the strategy to a greater degree (i.e., having a higher semantic clustering score on average) compared to their typical older adult counterparts. (**B**) Utilization of a semantic clustering strategy was defined by the extent to which participants were able to apply the strategy to a free recall trial called the “short delay” free recall trial. Semantic clustering scores during the short delay free recall trial are depicted on the *y* axis. (**C**) Utilization of a semantic clustering strategy was defined by the extent to which participants were able to apply the strategy to a free recall trial called the “long delay” free recall trial. Semantic clustering scores during the long-delay free recall trial are depicted on the *y* axis.

A one-way analysis of variance evaluating group differences for Sample 1 supported our hypothesis that Superagers would produce the semantic clustering strategy to a greater degree than typical older adults during the encoding trials (F(1, 38) = 17.81, *p* < 0.0001). Consistent with our prediction, the semantic clustering scores during the fifth encoding trial were higher for Superagers (M = 6.18, SD = 2.89) than for the typical older adult group (M = 2.15, SD = 3.05). These results were replicated in Sample 2 (F(1, 27) = 14.45, *p* < 0.0001). As in Sample 1, the semantic clustering scores during the fifth encoding trial were higher for Superagers (M = 7.25, SD = 0.77) than for typical older adults (M = 2.69, SD = 2.35) (see Figure 7A). In again leveraging the meta-analytic procedures described above, we summarized across the two studies and found that there was not a significant difference between the samples for both the effect size (z = −0.16, *p* = 0.56) and *p* value (z = −0.72, *p* = 0.23), and the combined estimate of the effect was 0.59, indicating a robust model effect across both samples.

Similarly, a one-way analysis of variance evaluating group differences for Sample 1 supported our hypothesis that Superagers would utilize the semantic clustering strategy to a greater degree than typical older adults during the short-delay free recall trial (F(1, 39) = 25.84, *p* < 0.0001). Consistent with our findings for production, the semantic clustering scores during the short-delay free recall trial were higher for Superagers (M = 6.31, SD = 1.86) than for the typical older adult group (M = 2.73, SD = 2.42). Again, these results were replicated in Sample 2 (F(1, 27) = 12.97, *p* < 0.01). As in Sample 1, the semantic clustering scores during the fifth encoding trial were higher for Superagers (M = 7.10, SD = 0.87) than for typical older adults (M = 3.05, SD = 2.19) (see Figure 7B). When summarizing across the two samples, we did not find a significant difference in the effects sizes for each model (z = −0.41, *p* = 0.33) nor in the *p*-values (z = −0.88, *p* = 0.19); the combined estimate of the effect across the two samples was 0.61.

In addition, we report semantic clustering during the long-delay free recall trial. Similar to the findings from our short-delay utilization analysis, a one-way analysis of variance evaluating group differences for Sample 1 supported our hypothesis that Superagers would utilize the semantic clustering strategy to a greater degree than typical older adults during the LDFR trial (F(1, 39) = 30.56, *p* < 0.0001). Consistent with our findings for production and short-delay utilization, the semantic clustering scores during the LDFR trial were higher for SA (M = 7.14, SD = 1.72) than for the TOA group (M = 3.39, SD = 2.37). These results were replicated in Sample 2 (F(1, 27) = 12.94, *p* < 0.01). As in Sample 1, the semantic clustering scores during the long delay recall trial were higher for Superagers (M = 7.10, SD = 1.19) than for typical older adults (M = 3.05, SD = 2.18) (see Figure 7C). When summarizing across the two samples, we did not find a significant difference in the effects sizes for each model (z = −0.37, *p* = 0.65) nor in the *p*-values (z = −1.09, *p* = 0.14), and the combined effect across both samples was 0.55.

## 4. Discussion

The goal of our study was to examine whether the production and utilization of a semantic clustering strategy was associated with better memory performance on the CVLT in two independent samples, with additional focus on the dynamics of strategy production and utilization in an unusual subset of elderly adults referred to as “Superagers.” In this study, we expanded on prior work on strategy use in verbal episodic memory test performance in aging, which has primarily focused on the use of semantic clustering during memory retrieval. Our results show that the production of an active semantic clustering strategy is associated with better verbal encoding, and that the utilization of this strategy is associated with better verbal memory retrieval performance. Furthermore, we demonstrated that this is one mechanism that appears to support Superagers’ superior memory performance relative to typical older adults. We showed that all these findings replicated in two independent samples. Thus, older adults with the best verbal episodic memory performance tend to spontaneously identify and capitalize on the opportunity to employ an active strategy to organize material to learn and remember it better and continued to utilize this strategy to organize recall during later test trials. This observation has potentially important implications for helping individuals supplement their memory abilities and potentially for cognitive rehabilitative efforts, since people can be taught to use such strategies to improve both encoding and recall of new information.

While a substantial number of studies have investigated semantic clustering strategy production during encoding, as it relates to retrieval performance, (e.g., [12,19,20,21,35]), very little work has been performed to examine how strategy production during encoding influences performance on the encoding trials themselves. Various studies have investigated strategy use in older adults by using a framework for “production” and “utilization” deficiencies [19,24,25], but in both cases, deficiency in the ability to recognize and maintain a strategy to augment memory performance is evaluated in the context of performance on the retrieval trials. Prior studies also reported that strategy production during encoding led to better memory performance, but in these studies, participants were explicitly instructed on the semantic clustering strategy during encoding [21,22,41]. Instead, we sought to investigate older adults’ spontaneous production of this strategy without cueing to understand how it supports encoding as well as retrieval. We believe that investigating the dynamics of strategy production and how it impacts the learning process itself is crucial, as interpretations of strategy use during recall without knowledge about strategy use during encoding would be limited, since strategy use during recall could reflect self-initiated strategy use during encoding, recall, or both [42]. Indeed, our findings suggest that in two separate samples of older adults, those who produce a strategy during the encoding trials achieved greater learning by the fifth and final encoding trial, suggesting that consistent production of a strategy during encoding is effective in optimizing the learning process.

In the present study, we found that those who utilized a semantic clustering strategy during the short and long delayed recall portion recalled more words during the delayed recall trial. Our findings are consistent with other behavioral studies which report that the use of a strategy can improve memory performance if the participant is able to recognize and apply the strategy during delayed recall [12,19,20,21]. However, the literature on the memory benefits of strategy utilization is mixed: some studies report a deficit in older adults’ ability to benefit from these strategies [14,42,43], while other studies report no age-related differences between older and younger adults (e.g., [30,31]). Our findings align with the former examples (e.g., [12,19,20,21]) in that, in addition to words learned in the preceding trials (e.g., recall on Trial 5 and short-delay trial for short and long-delay utilization, respectively), semantic clustering during the delayed free recall trial significantly improved memory performance across our two independent samples.

Finally, our findings also revealed that a subset of older adults called “Superagers”, whose superior memory performance during delayed recall trials distinguishes them from their typical older adult counterparts, showed greater production (i.e., higher semantic clustering scores during the fifth encoding trial) and utilization (i.e., higher semantic clustering scores during both delayed recall trials) relative to the rest of the sample. We previously reported that Superagers had higher semantic clustering scores relative to typical older adults during the long-delay free recall trial [10], but to our knowledge, no prior literature has looked at strategy production during encoding and utilization during delayed recall trials in Superagers. Prior literature on superior memory performance in the elderly is congruent with our finding that those who are high performers on memory tasks tended to employ a semantic clustering strategy (e.g., [44]). Furthermore, the spontaneous use of effective encoding strategies in older adults is correlated with superior processing speed [19,45], working memory capacity [14], and executive functioning [43,46,47]. Our data provide further support for the importance of semantic clustering strategies as a cognitive mechanism underlying superior memory performance in older adults.

Our results have implications for cognitive interventions in older adults [48]. These cognitive rehabilitation efforts aim to teach and re-teach strategies to older adults to support cognitive functions, with some strategies being optimized to support executive function (e.g., “Goal management training” [49], and others being optimized to support memory [50]). Several memory tools have been studied in older adults, such as internal strategies (associations like mnemonics) and external strategies (organizing one’s environment). One study showed that training older adults to use semantic encoding strategies increases memory performance as well as prefrontal and lateral temporal cortical activation during encoding [41]. Future research should investigate the generalization of semantic clustering as an efficacious strategy to scenarios in every-day life for the elderly.

This study has several limitations. Firstly, we only looked at the effect of semantic clustering on encoding and retrieval, but there are other types of active strategies, such as narratives, that may be similar or different to semantic clustering in terms of how they are produced and utilized throughout the encoding and retrieval portions of a memory assessment. Such strategies require self-report, whereas here we sought to use the verbal responses themselves on each trial as an explicit indication of semantic strategy use. Further, while we did find full replication of both the production and utilization models across Samples 1 and 2, there were some small differences across the two samples. The *R*^2^ values obtained from the adjusted models for both production and utilization indicated that Sample 1 participants tended to rely on both predictors in each model equally to improve performance, such that both predictors contributed equal amounts of explained variance in the combined model. However, for Sample 2, semantic clustering explained nearly double the variance explained by the other predictor in the model, for both production and utilization during short-delay free recall. One possible reason for these small discrepancies could be due to the makeup of the samples themselves; the first sample had a surprisingly large proportion of Superagers, while the second sample included a small proportion of Superagers (likely closer to the population-level representation). An additional limitation of our study is related to the distribution of sex across the two samples and among the Superager group versus the typical older adult group. Sex-specific differences in cognitive abilities are well established ([50,51], although these may be mediated by education differences; [52,53]) and higher proportions of female Superagers as opposed to male Superagers have been reported [54]. We observed this gender discrepancy in Sample 1, but not Sample 2 (see Table 1). To a certain extent, accounting for sex differences is difficult, given the nature of the Superaging sample; however, we have taken steps to ensure that the group differences seen in semantic clustering production and utilization are not being driven by sex differences between the two samples (see Supplementary Materials, Tables S2 and S3). Nonetheless, further research in this area should aim for better gender balance to determine whether our results are generalizable to a larger population of Superagers and typical older adults. Finally, both production and utilization models indicated that a combination of rote working memory and strategy use was optimal for memory performance. In future studies, collecting self-reported strategies and calculating indices of production and utilization across multiple strategies (i.e., serial clustering) in addition to the calculated semantic clustering scores would be one way to assess the extent to which the production and utilization of mixtures of strategies contribute to improved recall when a semantic clustering strategy appears to be absent or under-produced.

## 5. Conclusions

In this study, we show results which suggest that the production of an active semantic clustering strategy is associated with better verbal encoding, and that the utilization of this strategy is associated with better verbal memory retrieval performance in two independent samples of older adults. Furthermore, our results suggest that this may be one mechanism that appears to support Superagers’ superior memory performance relative to typical older adults. These results have implications for memory training interventions in older adults.

## Figures and Tables

**Table 1:** Demographic and Neuropsychological Characteristics.

	Sample 1		Sample 2	
Neuropsychological Measures	Superagers Mean (SD)	Typical Older Adults Mean (SD)	Superagers Mean (SD)	Typical Older Adults Mean (SD)
N	17	23	4	25
Age	67.47 (6.49)	66.44 (4.70)	79.50 (6.80)	68.48 (6.20)
Sex (% female)	71%	35%	50%	64%
Education (years)	17.21 (2.23)	16.22 (1.98)	18.00 (2.83)	19.76 (12.78)
Trail Making Test A (log)	1.45 (0.11)	1.43 (0.14)	1.92 (0.33)	1.82 (0.17)
Trail Making Test B (log)	1.76 (0.09)	1.78 (0.19)	2.07 (0.86)	1.95 (0.37)
CVLT Trial 1 (max 16)	9.71 (1.65)	7.27 (2.53)	8.00 (2.45)	6.72 (1.54)
CVLT Trial 5 (max 16)	14.71 (1.21)	12.00 (2.64)	14.75 (1.26)	12.36 (2.64)
CVLT Total Learning (Recall on Trials 1–5)	66.00 (5.16)	52.09 (10.99)	63.25 (7.09)	50.92 (7.55)
CVLT Short Delay Free Recall (max 16)	13.88 (1.72)	10.17 (3.28)	14.25 (0.96)	10.16 (2.70)
CVLT Long Delay Free Recall (max 16)	15.06 (0.97)	10.46 (3.16)	14.25 (0.50)	10.16 (2.78)

## Data Availability

The data that support the findings of this study are available from the corresponding authors, upon reasonable request. The data are not publicly available due to privacy and ethical restrictions.

## Supplementary Materials

The following supporting information can be downloaded at: https://www.mdpi.com/article/10.3390/brainsci14020171/s1, Table S1. Correlations between demographic variables and variables from the production models; Table S2. Correlations between demographic variables and variables from the utilization models; Table S3. Regression results for production models including demographic variables; Table S4. Regression results for short-delay utilization models including demographic variables; Table S5. Regression results for long-delay utilization models including demographic variables; Figure S1. Variance Inflation Factor plots for all predictors and demographic variables from production model; Figure S2. Variance Inflation Factor plots for all predictors and demographic variables from short-delay utilization model; Figure S3. Variance Inflation Factor plots for all predictors and demographic variables from long-delay utilization model. Figure S4. Summary of model R-squared values across production and utilization models.
