# Supplementary material for: Semantic Clustering during Verbal Episodic Memory Encoding and Retrieval in Older Adults: One Cognitive Mechanism of Superaging
Source: Brain Sci. 2024 Feb 8;14(2):171. doi: 10.3390/brainsci14020171 (PMC10886668; doi:10.3390/brainsci14020171)

Supplemental Materials

Table S1. Correlations between demographic variables and variables from the production models.

	Sample 1					Sample 2				
	Age	Education	Trial 1 Recall	Trial 5 Sem. Clustering	Trial 5 Recall	Age	Education	Trial 1 Recall	Trial 5 Sem. Clustering	Trial 5 Recall
Age		-0.05	0.04	0.12	0.23		-0.14	-.20	.18	-.05
Education	-0.05		0.43**	0.53**	0.33	-0.14		-.11	.19	.13
Sex	.097	0.11	0.42**	0.32*	0.38*	-.01	.01	.18	-.10	-.06

*p<.05, **p<.01, ***p<.001

Table S2. Correlations between demographic variables and variables from the utilization models.

	Sample 1						Sample 2					
	Age	Education	SDFR Recall	LDFR Recall	SDFR Clustering	LDFR Clustering	Age	Education	SDFR Recall	LDFR Recall	SDFR Clustering	LDFR Clustering
Age	1.00	-0.05	0.01	0.05	0.12	-0.03	1.00	-0.14		0.09	0.18	0.28
Education	-0.05	1.00	0.40	0.50*	0.42*	0.47*	-0.14	1.00		0.17	0.11	0.17
Sex	.097	0.11	.32*	.40*	.38*	.40*	.01	-.01	-.12	-.15	-.07	-.09

*p<.05, **p<.01, ***p<.001

Table S3. Regression results for production models including demographic variables.

	Sample 1: Recall on Trial 5		
Predictors	*Estimates*	*Conf. Int (95%)*	*P-Value*
Intercept	6.72	-0.85 – 14.28	0.080
Age	0.08	-0.04 – 0.20	0.167
Gender(F)	-0.39	-1.64 – 0.86	0.528
Education	-0.18	-0.52 – 0.16	0.293
Recall on Trial 1	0.33	0.02 – 0.64	**0.039**
Clustering on Trial 5	0.38	0.14 – 0.62	**0.002**
Observations	40		
R^2 / R^2 adjusted	0.594 / 0.534		

	Sample 2: Recall on Trial 5		
Predictors	*Estimates*	*Conf. Int (95%)*	*P-Value*
Intercept	16.68	8.08 – 25.27	**0.001**
Age	-0.11	-0.22 – -0.00	**0.042**
Gender(F)	-0.13	-1.70 – 1.43	0.863
Education	-0.01	-0.07 – 0.06	0.869
Recall on Trial 1	0.26	-0.20 – 0.72	0.246
Clustering on Trial 5	0.64	0.35 – 0.94	**<0.001**
Observations	29		
R^2 / R^2 adjusted	0.537 / 0.436		

Table S4. Regression results for short-delay utilization models including demographic variables.

	Sample 1: Recall on Short-Delay Trial		
Predictors	*Estimates*	*Conf. Int (95%)*	*P-Value*
Intercept	6.45	-0.35 – 13.25	0.062
Age	-0.10	-0.18 – -0.02	**0.018**
Gender(F)	0.17	-0.77 – 1.12	0.715
Education	0.03	-0.20 – 0.26	0.807
Recall on Trial 5	0.71	0.46 – 0.95	**<0.001**
Clustering on SDFR	0.50	0.27 – 0.72	**<0.001**
Observations	40		
R^2 / R^2 adjusted	0.848 / 0.825		

	Sample 2: Recall on Short-Delay Trial		
Predictors	*Estimates*	*Conf. Int (95%)*	*P-Value*
Intercept	1.59	-7.87 – 11.06	0.731
Age	0.02	-0.08 – 0.12	0.710
Gender(F)	-0.00	-0.06 – 0.06	0.975
Education	0.22	-1.14 – 1.57	0.743
Recall on Trial 5	0.43	0.06 – 0.80	**0.026**
Clustering on SDFR	0.65	0.26 – 1.03	**0.002**
Observations	29		
R^2 / R^2 adjusted	0.724 / 0.664		

Table S5. Regression results for long-delay utilization models including demographic variables

	**Sample 1: Recall on Long-Delay Trial**		
Predictors	*Estimates*	*Conf. Int (95%)*	*P-Value*
Intercept	-2.72	-9.35 – 3.92	0.411
Age	0.04	-0.04 – 0.12	0.359
Gender(F)	-0.42	-1.38 – 0.54	0.377
Education	0.19	-0.05 – 0.43	0.110
Recall on SDFR	0.72	0.50 – 0.93	**<0.001**
Clustering on LDFR	0.27	0.01 – 0.53	**0.041**
Observations	40		
R^2 / R^2 adjusted	0.855 / 0.834		

	**Sample 2: Recall on Long-Delay Trial**		
Predictors	*Estimates*	*Conf. Int (95%)*	*P-Value*
Intercept	3.42	-3.21 – 10.06	0.297
Age	-0.02	-0.10 – 0.07	0.664
Gender(F)	-0.02	-1.20 – 1.16	0.969
Education	0.01	-0.04 – 0.06	0.690
Recall on SDFR	0.65	0.39 – 0.90	**<0.001**
Clustering on LDFR	0.41	0.09 – 0.72	**0.014**
Observations	29		
R^2 / R^2 adjusted	0.795 / 0.750		

Figure S1. Variance Inflation Factor plots for all predictors and demographic variables from production model

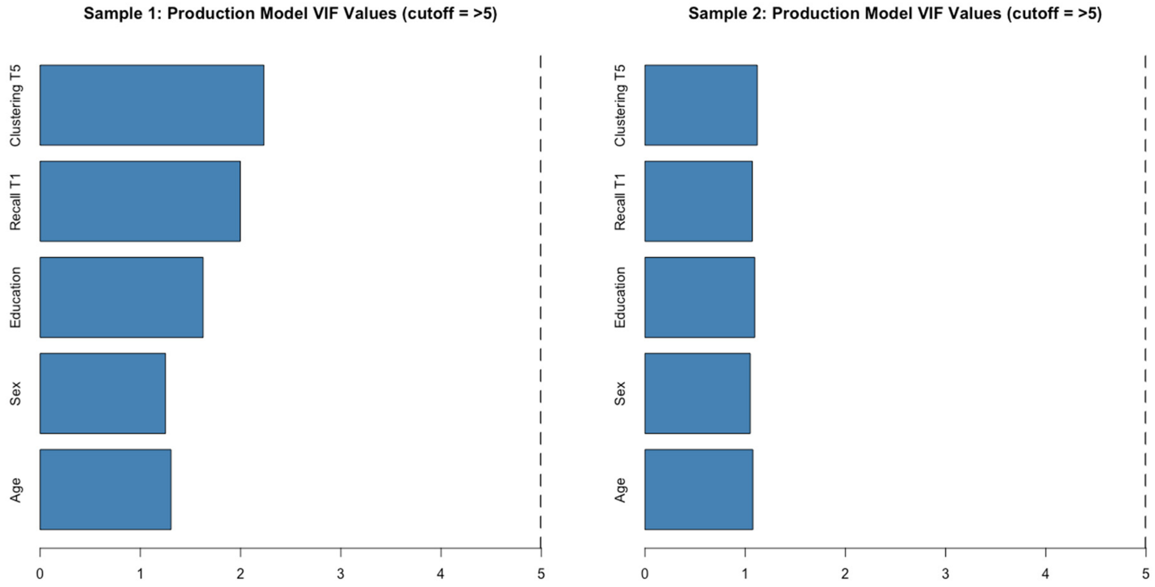

Figure S2. Variance Inflation Factor plots for all predictors and demographic variables from short-delay utilization model

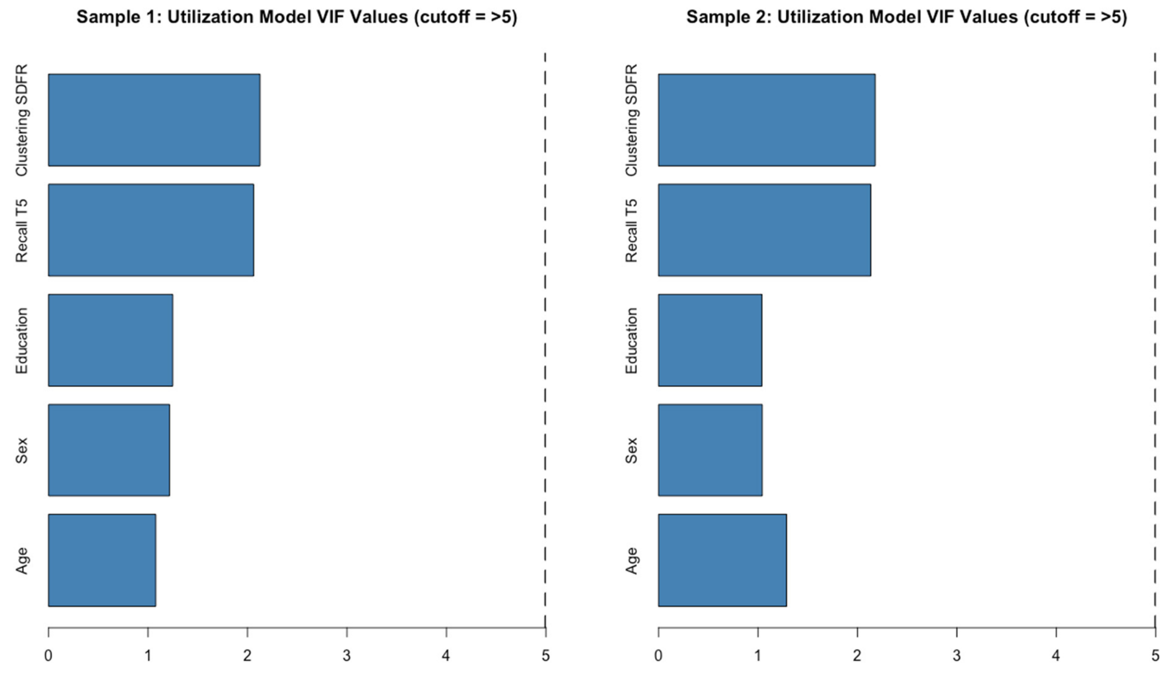

Figure S3. Variance Inflation Factor plots for all predictors and demographic variables from long-delay utilization mode

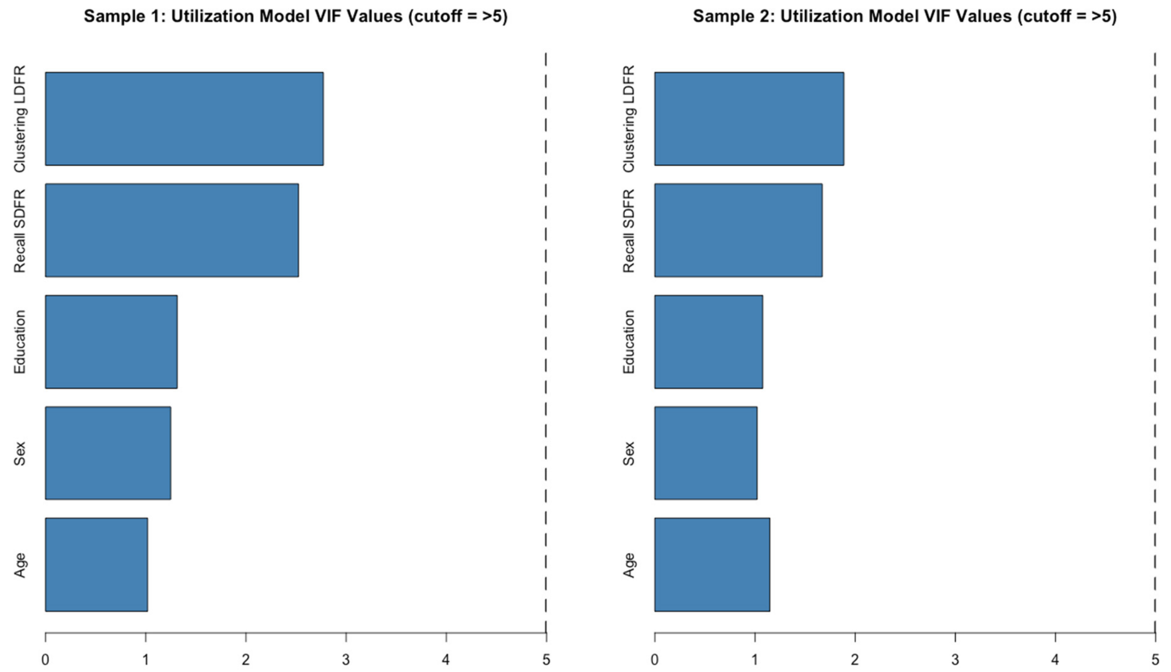

Figure S4. Summary of model R-squared values across production and utilization models.

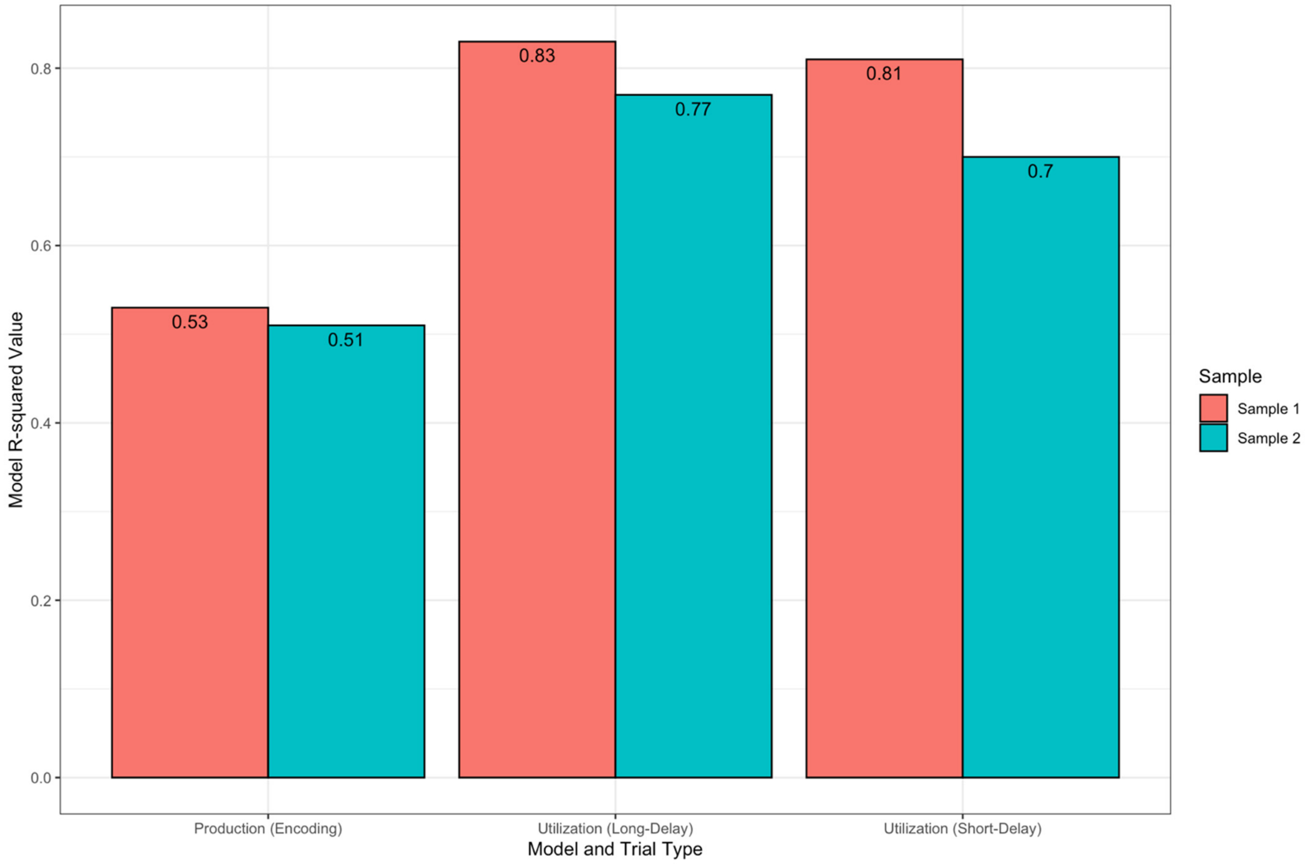

Supplement: Supplementary file 1 [file brainsci-14-00171-s001.zip › brainsci-2807847-supplementary.pdf]
